# Etiology and antimicrobial susceptibility patterns of bacteria causing pneumonia among adult patients with signs and symptoms of lower respiratory tract infections during the COVID-19 pandemic in Mwanza, Tanzania: a cross-sectional study

**DOI:** 10.1186/s41479-024-00137-9

**Published:** 2024-09-05

**Authors:** Johannes Rukyaa, Martha F. Mushi, Vitus Silago, Prisca Damiano, Katherine Keenan, Wilber Sabiiti, Matthew T. G. Holden, Jeremiah Seni, Stephen E. Mshana

**Affiliations:** 1 Department of Microbiology and Immunology, Weill Bugando School of Medicine, Catholic University of Health and Allied Sciences, P. O. Box 1464, Mwanza, Tanzania; 2 Division of Infection and Global Health, School of Medicine, University of St. Andrews, St. Andrews, KY16 9AL UK

**Keywords:** Bacteria, Pneumonia, Lower respiratory tract infection

## Abstract

**Background:**

Bacterial pneumonia is among the leading causes of morbidity and mortality worldwide. The extensive misuse and overuse of antibiotics observed during the Corona Virus Disease 2019 (COVID-19) pandemic may have changed the patterns of pathogens causing bacterial pneumonia and their antibiotic susceptibility profiles. This study was designed to establish the prevalence of culture-confirmed bacterial pneumonia and describe their antimicrobial susceptibility profile in adult patients who presented with signs and symptoms of lower respiratory tract infections (LRTIs) during the COVID-19 pandemic.

**Methodology:**

This hospital-based cross-sectional study was conducted from July 2021 to July 2022 at a zonal referral hospital and two district hospitals in Mwanza, Tanzania. Demographic and clinical data were collected using a standardized questionnaire. Sputum samples were processed by conventional culture followed by the identification of isolates and antibiotic susceptibility testing. Descriptive data analysis was performed using STATA version 15.0.

**Results:**

A total of 286 patients with a median age of 40 (IQR 29–60) years were enrolled in the study. More than half of the patients enrolled were females (52.4%, *n* = 150). The overall prevalence of bacterial pneumonia was 34.3% (*n* = 98). The majority of the bacterial pathogens isolated were Gram-negative bacteria (GNB) (61.2%, 60/98), with a predominance of *Klebsiella* spp., 38.8% (38/98), followed by *Streptococcus pyogenes* (21.4%, 21/98). Multi drug resistant (MDR) bacteria were detected in 72/98 (73.5%) of the isolates. The proportions of GNB-resistant strains were 60.0% (36/60) for ciprofloxacin, 60% (36/60) for amoxicillin, 60% (36/60) for amoxicillin, 68.3% (41/60) for trimethoprim-sulfamethoxazole and 58.3% (35/60) for ceftriaxone.

**Conclusion:**

One-third of the patients with signs and symptoms of LRTIs had laboratory-confirmed bacterial pneumonia with a predominance of Gram negative MDR bacteria. This calls for continuous antimicrobial resistance (AMR) surveillance and antimicrobial stewardship programs in the study setting and other settings in developing countries as important strategies for tackling AMR.

## Background

Lower respiratory tract infections (LRTIs) affect the trachea and alveolar sacs, resulting in bronchitis, acute exacerbations of chronic obstructive pulmonary disease, acute exacerbation of bronchiectasis and pneumonia [1]. Pneumonia is defined as acute inflammation of the parenchymal structure of the lung and can be classified based on the place of acquisition (community-acquired or hospital-acquired), causative agent (bacterial, viral, fungal, etc.) and mechanism (aspiration or ventilator-associated pneumonia) [2, 3].

Viruses led by Corona Virus Disease 2019 (COVID-19), are the leading causes of pneumonia globally, accounting for more than 770 million cases since 2019 [4]. Furthermore, viral infections predispose patients to secondary bacterial infections by overwhelming the immune system through cytokine storm and immune dysregulation, which may subsequently lead to an increased mortality and morbidity, as evidenced during the COVID-19 pandemic [5–7]. Data from South Africa revealed that up to 60% of severe acute respiratory syndrome-2 (SARS-CoV-2) patients who died had secondary bacterial infections [8].

Recent studies have reported an increase in the prevalence of antimicrobial overuse and misuse aimed at the treatment of COVID-19 and its related symptoms [9]. This was due to the lack of immediate and appropriate effective treatments for COVID-19, a viral disease that cannot be treated with antibiotics [10]. Moreover, the reallocation of resources and deviation of the focus from antimicrobial stewardship programs toward COVID-19 mitigation were among the driving factors of the misuse of antibiotics [11, 12]. This leads to increased antimicrobial resistance (AMR) among bacterial pathogens that cause common infections [9].

In the study, wide dispensing of antibiotics without prescriptions was observed during the COVID-19 pandemic for COVID-19-like symptoms [13]. This is due to a lack of antimicrobial stewardship, especially during the COVID-19 pandemic, in Tanzania and other low- and middle-income countries [13].

Globally, studies have documented an increase in AMR after the COVID_19 pandemic, which was associated with the misuse and overuse of antibiotics for the treatment of COVID-19-like symptoms [14]. The picture might be different in Tanzania due to variation in treatment preference during the pandemic because a large pool of individuals opted to use herbal medication and steam therapy as treatment options [15, 16].

A study by Kishimbo et al., 2020 in a similar setting during the pre-COVID-19 pandemic reported a predominance of Gram negative multidrug resistant (MDR) bacteria causing bacterial pneumonia caused by *Klebsiella* spp. [17].

The COVID-19 pandemic may have changed the patterns of bacteria causing pneumonia while accelerating the progression of AMR and the increase in MDR bacteria in our setting. Therefore, this study was designed to establish the pattern of culture confirmed bacterial pneumonia and its antimicrobial susceptibility pattern among patients with signs and symptoms of LRTI’s during the COVID-19 pandemic.

## Methods

### Study design, duration, population and setting

This hospital-based cross-sectional study was conducted between July 2021 and July 2022 among adult patients aged ≥ 18 years with signs and symptoms of lower respiratory tract infections (LRTIs) admitted to and admitted to Sengerema District Hospital, Nyamagana District Hospital and Bugando Medical Centre (BMC) in Mwanza, Tanzania.

### Sample size estimation and selection criteria

The minimum sample size of 285 was obtained by the Kish-Leslie formula [18] using a prevalence of bacterial pneumonia of 20.4% from a previous study by Kishimbo et al., 2020 [17]. The study enrolled a total of 286 adult patients who presented with a productive cough and at least two of the following symptoms: fever, axillary temperature > 37.5 °C or hypothermia < 36.1 °C, chest pain/discomfort or dyspnea, infiltrates demonstrated on chest radiography, auscultatory findings consistent with pneumonia (altered breath sounds and/or localized rales), shortness of breath or difficulty breathing, nausea or vomiting, loss of taste or smell, sore throat, muscle or body aches.

### Data and sample collection

Sociodemographic (e.g., age, sex and place of residence) and clinical (e.g., signs and symptoms) data were collected from the enrolled patients via a standardized questionnaire using Epicollect5 [19]. Patients were instructed to expectorate into sterile, wide-mouthed screw-capped and leak-proof specimen containers. Sputum samples were collected on spot and transported to the microbiology laboratory for processing within 2 h of collection in a cool box.

### Laboratory procedures

#### Gram stain

A portion of the sputum was selected using a sterile wire loop and used to create a thin smear on a clean labeled microscopy slide (BENOYLAB, Jiangsu Benoy Lab Instrument Co., Ltd. Jiangsu China). The smear was air-dried and then heat-fixed on an electric hotplate, followed by Gram staining (Gram Staining Kit, Himedia, Bottal, India) [20]. The quality of the sputa was assessed using the criteria of *Bartlett et al.*. [21]. A sputum with a Q-score greater than 1 was considered acceptable and termed good quality, while a score of “0” or “–” was considered poor quality [21]. All sputum samples were processed for culture so as not to miss the isolation of bacteria that elicit a low neutrophil response, such as *H. influenzae* [22].

#### Culture for the isolation of bacterial pathogens causing bacterial pneumonia

Sputum was directly inoculated onto chocolate agar (OXOID, Hampshire, United Kingdom), blood agar (OXOID, Hampshire, United Kingdom) and MacConkey agar (OXOID, Hampshire, United Kingdom) using a 10 µl loop and incubated for 18–24 h at 35–37 °C. Blood agar and chocolate agar plates were incubated in candle jars to achieve 5–7% CO_2_ for the isolation of fastidious bacterial pathogens such as *H. influenzae* [23].

#### Biochemical and physiological identification testing for identification of isolated bacterial pathogens

The identification of the bacterial pathogens was performed based on the growth characteristics on blood agar, MacConkey agar and chocolate agar, secondary Gram stain, and in-house biochemical tests, such as Christensen urea agar, triple sugar iron (TSI) agar, Simmons citrate agar, sulphur indole motility (SIM) agar, and oxidase for gram-negative bacteria, whereas catalase, coagulase, bile aesculin, optochin, novobiocin, and DNase were used for gram-positive bacteria [20, 23].

### Antimicrobial susceptibility testing

A single colony of bacteria from a fresh pure culture plate was emulsified in sterile normal saline to achieve a concentration equivalent to 0.5 McFarland turbidity solution. A sterile cotton swab was then used to obtain bacteria from the suspension, and the swab was then squeezed against the wall of the tube to remove excess fluid before being seeded uniformly onto a Muller-Hinton agar (OXOID, Hampshire, United Kingdom) plate. Antibiotics of the right potency were placed on agar to test for susceptibility patterns using Kirby Bauer’s disc diffusion method [24] as guided by the Clinical Laboratory Standard Institute (CLSI) 30th edition M100 document, 2020 [25]. Antibiotic discs for Gram-positive bacteria included ampicillin (10 µg), cefoxitin (30 µg), trimethoprim-sulfamethoxazole (1.25/23.75 µg), ciprofloxacin (5 µg), erythromycin (15 µg), clindamycin (2 µg), vancomycin (30 µg), linezolid (30 µg), gentamicin (10 µg), clindamycin (2 µg), and tetracycline (30 µg), whereas those for Gram-negative bacteria included ceftriaxone (30 µg), cefepime (30 µg), ciprofloxacin (5 µg), ceftazidime (30 µg), piperacillin-tazobactam (100/10 µg), amoxicillin/clavulanate (20/10 µg), trimethoprim-sulfamethoxazole (1.25/23.75 µg), meropenem (10 µg), amikacin (30 µg), gentamicin (10 µg) and tetracycline (30 µg).

All *S. aureus *strains with a zone of inhibition on a cefoxitin (30 µg) disc ≤ 21 mm were regarded as methicillin-resistant *S. aureus* (MRSA); inducible clindamycin resistance was tested by observing the blunting of the zone of inhibition around the clindamycin disc placed adjacent to the erythromycin disc. For Gram-negative bacteria, the extended-spectrum beta-lactamase (ESBL) phenotype was confirmed using the combined disc method with 30 µg cefotaxime and/or 30 µg ceftazidime (with and without 10 µg clavulanic acid) [25].

### Quality control

Control strains of *Streptococcus pneumoniae* (ATCC 49,619), *Haemophilus influenzae* (ATCC 49,247/49,766), *Staphylococcus aureus* (ATCC 25,923) and *Escherichia coli* (ATCC 25,922) were used for quality control of the culture media and the antibiotic discs [25]. MDR was confirmed when resistance to three or more classes of antibiotics was observed among the isolated bacterial pathogens [26].

### Data management and analysis

Excel data sheet was extracted from Epi-collect- 5 software^®^ and then, laboratory data were also added into the Microsoft Excel for cleaning and coding. Data was then transferred to STATA version 15 (College Station, Texas, USA) for analysis. Continuous data was summarized using a medium with an inter-quartile range (IQR). Categorical data were summarized using proportions (percent). Pearson chi squared test (or Fisher’s exact where applicable) was used to assess the distribution of categorical variables against culture positivity, and a p-value of less than 0.05 with 95% confidence interval was considered as statistically significant.

### Ethical considerations

This study was approved by the CUHAS/BMC Research Ethics and Review Committee with the certificate number CREC/607/2022, and the study also received further clearance from the National Health Research Ethics Review Committee (NATHREC) with the certification number NIMR/HQ/R.8a/Vol.IX/2831. Permission to conduct the study was obtained from the BMC, Sengerema district hospital and Nyamagana district hospital authorities. Informed consent was obtained from all patients.

## Results

### Sociodemographic characteristics of the study participants

This study enrolled a total of 286 adult patients with signs and symptoms of LRTIs. Half of the enrolled patients were female (150, 52.4%). The median age of the study participants was 40 (IQR 29–60) years, with approximately two-thirds of the study participants being married (188, 65.7%) and 179 (62.6%) employed, as shown in Table 1.

**Table 1:** Sociodemographic data of the enrolled patients

Variables	Median (IQR)/Frequency (%) *N* = 286
**Age(years)**	40 (IQR 29–60)
**Sex**	
Female	150 (52.4%)
Male	136 (47.6%)
**Education level**	
Primary	25 (8.7%)
No schooling	101 (35.3%)
Secondary	77 (26.9%)
Tertiary	83 (29.0%)
**Marital status**	
Married	188 (65.7%)
Not married	98 (34.2%)
**Occupation**	
Employed	179 (62.6%)
Not employed	107 (37.4%)
**Hospital level**	
District	103 (36.0%)
Tertiary	183 (64.0%

The majority of the patients (203 [71.0%]) had a history of antibiotic use within two weeks prior to specimen collection. Having visited the hospital in the past one year prior to specimen collection was significantly associated with a positive sputum culture (95% CI: *p* < 0.031), the most commonly reported comorbidity was HIV/AIDS 24 (35.5%). Among all the study participants, 148 (51.7%) claimed to use past experience as a source of information for antibiotic use, as shown in Table 2.

**Table 2:** Clinical information for the enrolled patients

Variables	Frequency (%)	Culture results Positive(%)		*P* value
		Positive (%)	Negative (%)	
**Comorbidity**				
Yes	68 (23.8%)	19 (27.9)	49 (72.1)	0.100
No	218 (76.2%)	82 (37.6)	136 (62.4)	
**Type of comorbidity**				
HIV/AIDS	24 (35.3%)	5 (20.8)	19 (79.2)	
Diabetes	9 (13.2%)	7 (77.8)	2 (22.2)	
Others **	15 (22.1%)	11 (73.3)	4 (26.7)	
High blood pressure	20 (29.4%)	14 (70.0)	6 (30.0)	
**Antibiotic use within 2 weeks of** specimen collection				0.742
Yes	203 (71.0%)	69 (34.0)	134 (66.0)	
No	83 (29.0%)	30 (36.1)	53 (63.9)	
**Information on antibiotic use**				0.684
**Advice from doctor or HCW**				
Yes	260 (90.9%)	180 (69.2)	80 (30.8)	
No	26 (9.1%)	17 (65.4)	9 (34.6)	
**Advice from chemist/drug seller**				0.332
Yes	223 (78.0%)	110 (49.3)	113 (50.7)	
No	63 (22.0%)	33 (52.4)	30 (47.6)	
**Based on past experience**				0.216
Yes	148 (51.7%)	59 (39.9)	89 (60.1)	
No	138 (48.3%)	50 (36.2)	(63.7)	
**Hospital visits within a year**				0.031
Yes	176 (61.5%)	50 (28.4)	126 (71.6)	
No	110 (38.5%)	48 (43.6)	62 (56.4)	
**Hospital status**				0.394
Inpatients	97 (33.9%)	30 (30.9)	67 (69.1)	
Outpatients	189 (66.1%)	68 (36.0)	121 (64.0)	

** Cancer (2), stroke (1), heart disease (2), arthritis (2), mental health problems (2), muscular problems (2), wounds (2) and peptic ulcer disease (2)

Of the 203 study participants who had used antibiotics two weeks prior to sample collection, 52 (25.6%) had used antibiotics that were not indicated in the Tanzanian standard treatment guidelines (STGs) for the treatment of bacterial pneumonia. Azithromycin, ampicillin-cloxacillin and ciprofloxacin were the most frequently used antibiotics (46.8%, 24% and 16.3%, respectively), as shown in Table 3.

**Table 3:** Antibiotics used by patients for the treatment of bacterial pneumonia and their STG **indications**

Antibiotic	Frequency	Percentage (%)	STG indication for pneumonia treatment
Ampicillin- cloxacillin	49	24	INDICATED
Augmentin	7	3.4	INDICATED
Azithromycin	95	46.8	INDICATED
Metronidazole	8	3.9	NOT INDICATED
Tetracycline	4	2.0	NOT INDICATED
Ciprofloxacin	33	16.3	NOT INDICATED
Trimethoprim/Sulphamethoxazole	7	3.4	NOT INDICATED
**TOTAL**	**203**	**100%**	

### Prevalence of bacterial pneumonia

**Fig. 1:**
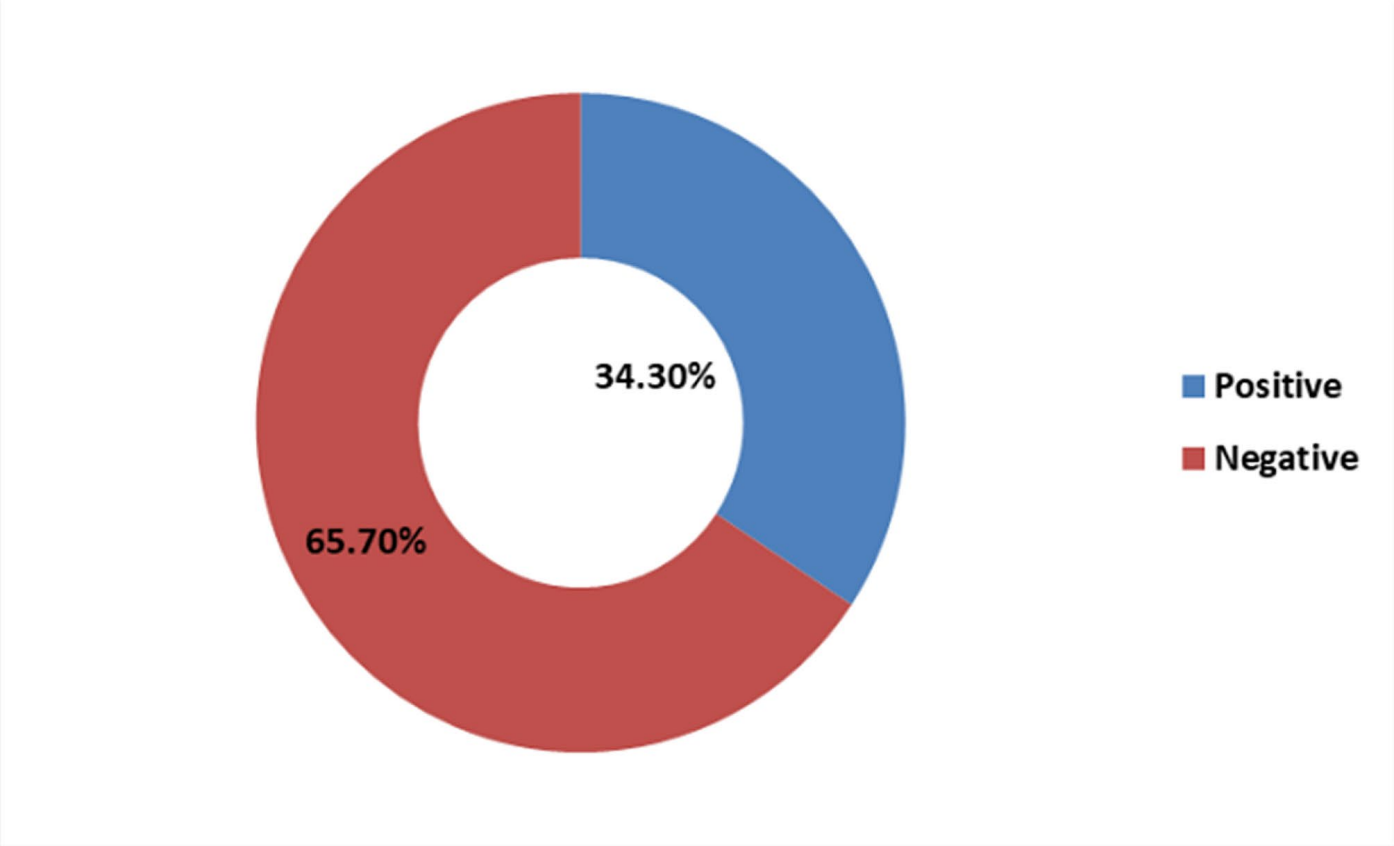
Culture results for sputum samples

Out of 286 nonrepetitive sputum samples processed, 34.3% (98/286) had a positive culture for pathogenic bacteria, with a 95% CI of 24-43% (Fig. 1). Of the 286 non-repetitive sputum samples, only 153 (53.5%) were of good quality according to the Bartlett scoring criteria; 96 (62.7%) of the good-quality sputum samples were culture positive for pathogenic bacteria, while only 2 (1.5%) of the poor-quality sputum samples were culture positive (*P* < 0.001).

### Bacterial pathogens causing pneumonia per health care facility

Out of 183 sputum samples from patients attending tertiary hospitals, 64 (35%) had a positive culture for pathogenic bacteria, whereas of the 103 sputum samples from patients attending district hospitals, only 34 (33%) had a positive culture for pathogenic bacteria (*p* = 0.939) (Table 4).

**Table 4:** Distribution of bacterial isolates from culture-positive sputum (*N* = 98)

Isolates	Tertiary	District	Total *N* = 98 Frequency (%)
*Klebsiella* spp.	26	12	38 (38.8%)
*S. pyogenes*	10	11	21 (21.4%)
*E. coli*	12	3	15 (15.3%*)*
Other gram-positive bacteria *	14	3	17 (17.3%)
Other gram-negative bacteria **	2	5	7 (7.4%)
Total	64 (65.3%)	34 (34.7%)	

*Enterococcus spp. (6), *S. aureus* (6), *S. pneumoniae* (5), ** *E. cloacae* (3), *Acinetobacter* spp. (1), *P. aeruginosa* (1) and *P. mirabilis* (2)

### Resistance pattern for Gram-negative bacteria

**Fig. 2:**
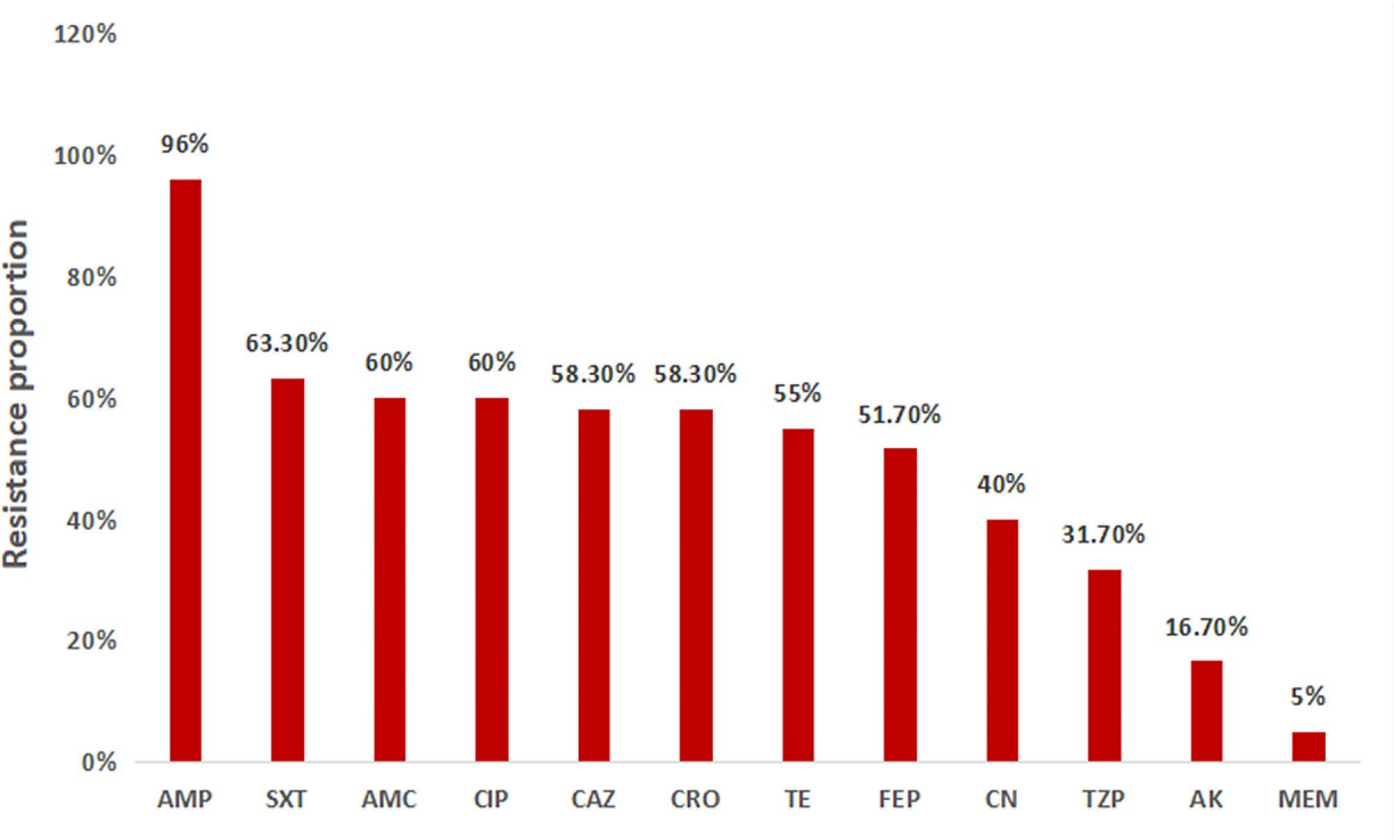
Antibiotic resistance proportions of gram-negative bacteria (*N* = 60). *Ampicillin was not applicable for testing against 35-gram-negative isolates. Keywords: AMP = ampicillin, CIP = ciprofloxacin, SXT = trimethoprim/sulfamethoxazole, CN = gentamicin, TE = tetracycline, CRO = ceftriaxone, TZP = piperacillin/tazobactam, AMC = amoxicillin/clavulanate, MEM = meropenem, AK = amikacin, CAZ = ceftazidime, FEP = cefepime.

A total of 60 Gram-negative bacteria were subjected to AST; GNB strains with resistance to ampicillin (*n* = 25)*, 24/25 (96.0%) had the highest proportion of resistance, followed by those with resistance to trimethoprim-sulfamethoxazole (41/60, 68.3%), amoxicillin/clavulanate (38/60, 63.3%), and ciprofloxacin (36/60, 60.0%). Meropenem and amikacin had the lowest proportions of resistance, at 3/60 (5.0%) and 10/60 (16.7%), respectively, as shown in Fig. 2.

Overall, the proportions of antibiotic-resistant *E. coli* were greater than those of *Klebsiella* spp., as shown in Table 5. Among the 38 *Klebsiella* spp., 21 (51.3%) were resistant to ceftazidime and ceftriaxone, while 12/15 (80.0%) were resistant to * E. coli*, as shown in Table 5.

**Table 5:** Antibiotic resistance patterns of gram-negative bacterial isolates

Antibiotic	*Klebsiella* spp. *N* = 38	E. coli*N* = 15	Other GNB *N* = 7
AMP	N/A	15 (100%)	4 (100%) *
CIP	21 (55.3%)	11 (73.3%)	4 (57.1%)
SXT	26 (68.4%)	14 (93.3%)	1 (14.3%)
CN	12 (31.6%)	10 (66.7%)	1 (14.3%)
TE	18 (47.4%)	12 (80.0%)	3 (42.9%)
TZP	10 (26.3%)	6 (40.0%)	3 (42.9%)
AMC	22 (57.9)	13 (86.7%)	3 (42.9%)
MEM	2 (5.3%)	0 (0.00%)	1 (14.3%)
AK	6 (15.8%)	4 (26.7%)	0 (0.0%)
FEP	18 (47.0%)	12 (80.0%)	1 (14.3%)
CAZ	21 (55.3%)	12 (80.0%)	2 (28.6) %
CRO	21 (55.3%)	12 (80.0%)	2 (28.6) %

Keywords: GNB = gram-negative bacteria, other GNB *Acinetobacter* spp. *(1), P. aeruginosa (2) and P. mirabilis (1), E. cloacae (3)*, AMP = ampicillin, CIP = ciprofloxacin, SXT = trimethoprim/sulfamethoxazole, CN = gentamicin, TE = tetracycline, CRO ceftriaxone, TZP = piperacillin/tazobactam, AMC = amoxicillin/clavulanate, MEM = meropenem, AK = amikacin and erythromycin, CAZ = ceftazidime, FEP = cefepime. * Not tested for *P. aeruginosa, Acinetobacter* spp

### Antibiotic resistance patterns for Gram-positive bacteria

**Fig. 3:**
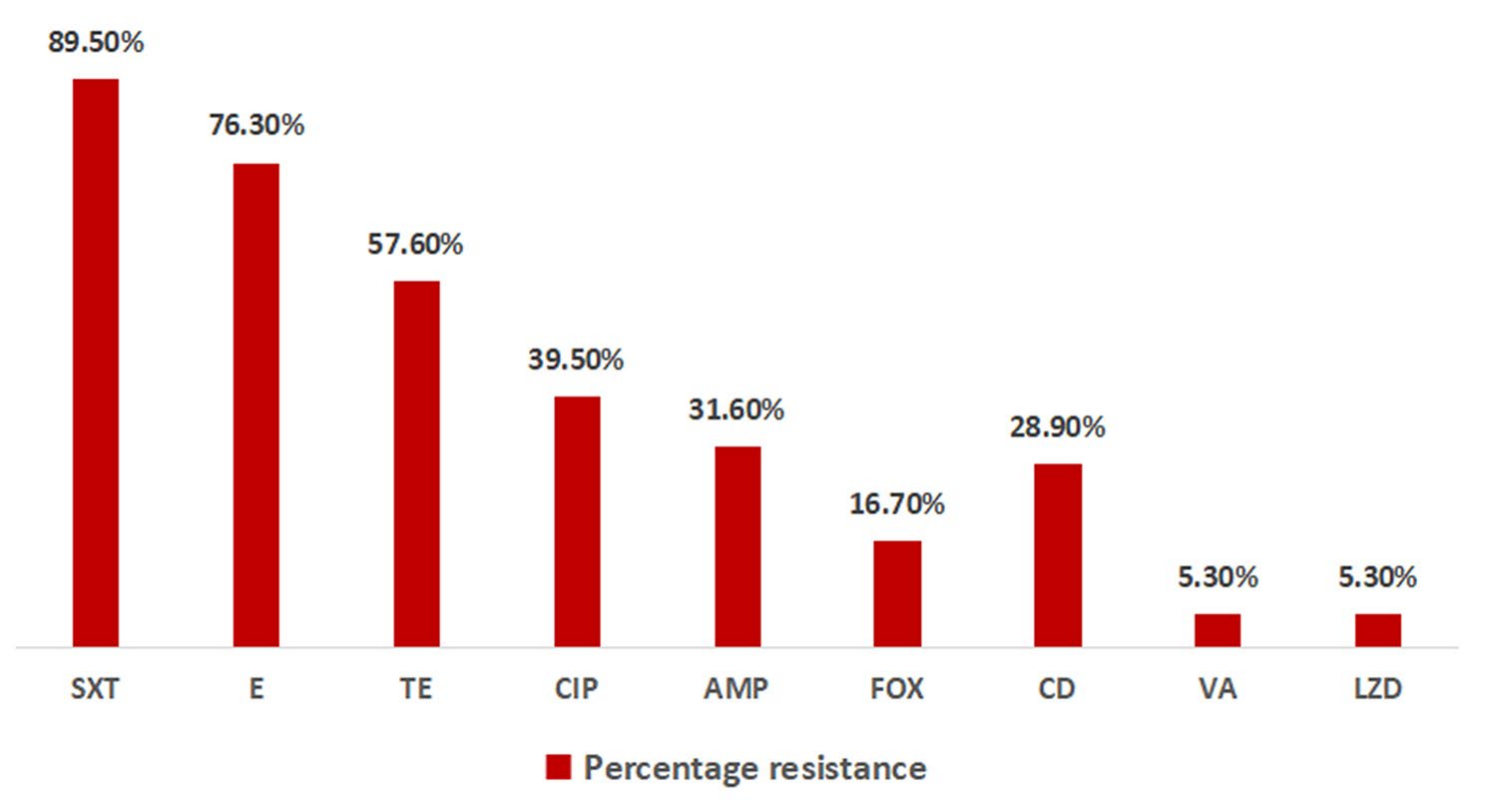
General antibiotic resistance pattern for Gram-positive bacteria, *N* = 38. Keywords: AMP = ampicillin, CIP = ciprofloxacin, SXT = trimethoprim/sulfamethoxazole, CN = gentamicin, TE = tetracycline, E = erythromycin, FOX = cefoxitin, LZD = linezolid, VA = vancomycin.

Among the Gram-positive bacteria (*n* = 38), 34/38 (89.5%) had resistance to trimethoprim/sulfamethoxazole, 29/38 (76.3%) had resistance to erythromycin, and 22/38 (57.9%) had resistance to tetracycline (Fig. 3).

*S. pyogenes* showed high proportions of resistance to erythromycin (18/21, 85.7%), tetracycline (76.2%, 16/21) and ciprofloxacin (57.1%, 12/21), whereas for other gram-positive strains, erythromycin (11/17, 64.7%) and ampicillin (10/17, 58.8%) had the highest resistance proportions, as shown in Table 6. All the isolated *S. pneumoniae* strains were resistant to erythromycin, tetracycline and trimethoprim/sulfamethoxazole. None of the isolated *S. pyogenes* or *S. aureus* strains were ICR positive. All *S. pneumoniae* strains were sensitive to penicillin, and only 16.6% (1/6) of the *S. aureus* strains were MRSA.

**Table 6:** Antibiotic resistance patterns of individual Gram-positive bacterial isolates

Antibiotics	*S. pyogenesN* = 21	Other Gram-positive bacteria *N* = 17
AMP	0 (0.00%)	10 (58.8%)
CIP	12 (57.1%)	3 (17.6%)
FOX	N/A	1 (16.7%) *
E	18 (85.7%)	11 (64.7%)
CD	5 (23.8%)	6 (35.3%)
LZD	2 (9.5%)	0 (0.00) **
VA	1 (4.8%)	1 (5.8%)
TE	16 (76.2%)	6 (35.3%)
SXT	21 (100%)	13 (76.5%)
CN	14 (66.7%)	2 (18.2%) **

*FOX was only tested for *S. aureus*, ** LZD and CN were not tested for *Enterococcus* spp. Others: *S. pneumoniae* (5), *S. aureus, and Enterococcus* spp. (6). Keywords: AMP = ampicillin, CIP = ciprofloxacin, SXT = trimethoprim/sulfamethoxazole, CN = gentamicin, TE = tetracycline, E = erythromycin, FOX = cefoxitin, LZD = linezolid, VA = vancomycin

### Multidrug-resistant bacteria

A high proportion of multidrug-resistant (MDR) bacteria was detected in this study; of the 98 isolated bacterial pathogens, 72/98 (73.5%) were MDR, with *Klebsiella* spp. being the predominant contributor. Among those isolated at the district hospital level (*n* = 34), 17 (50.0%) were MDR, while among those isolated at the tertiary hospital level (*n* = 64), 55 (86.0%) were MDR (*p* = 0.0019).

## Discussion

In this laboratory-based cross-sectional study, the prevalence of microbiologically confirmed bacterial pneumonia during the COVID-19 pandemic was 34.3%. These findings are significantly greater than those of a previous study before COVID-19 pandemic by Kishimbo et al. in similar settings, which reported a prevalence of 20.4% [27](*p* < 0.005). This increase may be attributed to the COVID-19 pandemic, which has been reported to predispose patients to secondary bacterial infections by overwhelming their immunity via cytokine overproduction and immune system dysregulation, leading to poor protection against bacterial pneumonia and impeding proper mucociliary clearance of potential pathogenic bacteria through mucociliary killing [28, 29].

Overall, 71% of the participants reported having used antibiotics two weeks prior to specimen collection, with azithromycin, ampicillin-cloxacillin and ciprofloxacin being the most frequently used antibiotics. This may have been accelerated by the ongoing COVID-19 pandemic, which has led to a surge increase in the use of antibiotics as a treatment and management option for COVID-19-like symptoms [30]. The observed use of azithromycin and ampicillin-cloxacillin by the study participants contradicts the current Tanzanian standard treatment guidelines for the treatment of bacterial pneumonia [31]. This is further supported by the fact that 25.6% of the study participants who used antibiotics for the treatment of bacterial pneumonia used antibiotics that were not indicated by the Tanzanian STG. This may have been caused by the current COVID-19 pandemic, which has led to an increase in the overprescription, misuse and overuse of antibiotics in efforts to combat and mitigate the outbreak [32], as supported by findings by Olamijuwon et al., 2021, who reported high rates of mismatched prescription of antibiotics contrary to those of the STG [13].

This study revealed a high proportion of azithromycin use among study participants two weeks prior to specimen collection, at a proportion of 46.8%; this may have been accelerated by the fact that azithromycin was among the antibiotics preferred during the pandemic as a drug of choice in the management of the COVID-19 pandemic [33, 34]. Similar to a previous report by Kishimbo et al., this study revealed the predominance of Gram-negative bacteria led by Klebsiella spp. [27]. These results show that the epidemiology of bacterial pneumonia with regard to its etiology has not changed in our setting, regardless of the presence of COVID-19; however, the findings differ from those in other geographical settings, which have indicated that *S. pneumoniae* is the leading cause of bacterial pneumonia [35]. This further proves why the use of STG for the treatment of bacterial pneumonia should be structured based on local findings.

Our study revealed that sputum samples with a good Bartlett quality score had a greater probability of having pathogenic bacteria than those with a poor quality score, which was in agreement with the findings of Kishimbo et al [27]. Sputum culture is a good tool for the diagnosis of bacterial pneumonia if collected and processed properly [36]; however, sputum quality determines the quality of the culture results, with good quality sputa having the best culture yield for pathogenic bacteria compared to poor quality sputa [37]. Therefore, clinicians and laboratory scientists should encourage the collection of good-quality sputum for the diagnosis of bacterial pneumonia.

Our findings revealed a high proportion of resistant Gram-negative bacteria to ciprofloxacin, trimethoprim-sulfamethoxazole, ceftriaxone and ceftazidime. These findings are different from those reported by Kishimbo et al. The data reported in this study revealed an increase in the proportion of resistance of the *Klebsiella* spp. to ciprofloxacin (55.3% vs. 17.4%), gentamicin (31.6% vs. 26.1%), and trimethoprim/sulfamethoxazole (68.4% vs. 43.5%) [27]. The increase in the prevalence of resistance may be attributed to the overuse of antibiotics during the COVID-19 pandemic, as observed in this study, where the majority of the participants used antibiotics within two weeks prior to sample collection [13].

The isolated Gram-positive bacteria showed high proportions of resistance to tetracycline, ciprofloxacin and erythromycin; these findings are different from those reported by Kishimbo et al., who reported comparatively lower proportions of *S. pyogenes* resistance to erythromycin [27]. The observed misuse and overuse of azithromycin during the COVID-19 pandemic, as reported by Sagenda et al., in a study in which the use of azithromycin in Tanzania increased by 163.7% after COVID-19 [30].

We observed high proportions of MDR pathogens in this study from both district and tertiary hospitals, which may be attributed to the high levels of antibiotic overuse during the COVID-19 pandemic leading to resistance development due to increased antibiotic pressure [38]. Furthermore, there was a greater proportion of MDR pathogens isolated from tertiary hospitals than from district hospitals, possibly because patients attending tertiary hospitals are more exposed to antibiotics than are those attending lower hospitals [39]. These findings are supported by those from Brazil, Italy and the United Kingdom, which reported the influence of COVID-19 on the increase in the overuse and misuse of antibiotics, leading to an increase in MDR among bacterial pathogens [40–42].

This study revealed high proportions of strains resistant to erythromycin, ciprofloxacin and 3rd -generation cephalosporins, which are included in the AWaRE WHO classification category of 2021. [43], this calls for continuous surveillance and antimicrobial stewardship programmes.

### Limitations

Failure to isolate or detect pathogens known to cause atypical pneumonia, such as *Legionella pneumophila* and *Chlamydia pneumoniae*, may have led to underestimation of the true magnitude of bacterial pneumonia. In addition, due to shortcomings of the clinical information in the patients file, the correlation with X rays findings, clinical information and microbiological findings was not done. Furthermore, this study did not include information on the COVID 19 status of participants and only few patients presented with comorbidities data.

## Conclusion

One-third of the patients with signs and symptoms of LRTIs had laboratory-confirmed bacterial pneumonia with a predominance of multidrug-resistant Gram-negative bacteria. The observed high proportions of resistance among Gram-negative bacteria to third-generation cephalosporins and ciprofloxacin call for continuous AMR surveillance to obtain data that can guide empiric antibiotic treatment.

## Data Availability

No datasets were generated or analysed during the current study.

## Abbreviations

AMR: Antimicrobial resistance
ATCC: American Type Culture Collection
BMC: Bugando Medical Centre
CLSI: Clinical Laboratory Standard Institute
COVID-19: Coronavirus 2019
CUHAS: Catholic University of Health and Allied Sciences
ESBL: Extended spectrum beta lactamase
LRTIs: Lower respiratory tract infections
MDR: Multidrug resistant
MRSA: Methicillin-resistant S. aureus
SARS-CoV-2: Severe acute respiratory syndrome-2
SIM: Sulphur indole motility
STG: Standard treatment guideline
TSI: Triple sugar iron
